# Exploring English for medical purposes (EMP) teacher cognition in the Chinese context

**DOI:** 10.3389/fpsyg.2022.1003739

**Published:** 2022-09-28

**Authors:** Zhongkai Cao, Zaihong Zhang, Ya Liu, Liping Pu

**Affiliations:** ^1^School of Foreign Languages, Huazhong University of Science and Technology, Wuhan, China; ^2^School of Foreign Languages, Hubei University of Chinese Medicine, Wuhan, China; ^3^Soochow College, Soochow University, Suzhou, China

**Keywords:** EMP teacher, teacher cognition, teacher characteristics, EMP teaching, Chinese context

## Abstract

It has been a growing trend in Chinese universities to shift from English for general purposes (EGP) to English for specific purposes (ESP) teaching. Against this background, large groups of teachers previously engaged in teaching EGP have become or are becoming ESP teachers, which means a complex process of learning for subject-specific information, transforming teaching practices and constructing new identities. Despite this, very little has been written about the ESP teacher cognition (TC) of language teaching or the factors influencing this shift in teaching. This study involved English for Medical Purposes (EMP) teachers in Chinese universities as participants, and a scale of EMP TC with 31 items was developed on the basis of questionnaire results. Data from exploratory factor analysis revealed six dimensions of the scale, namely, teacher attitude, teacher belief, teacher learning, teacher support, role identification, and teacher practice—that combine to constitute and influence EMP TC. While the identity factor has attracted wide attention in ESP teacher research, other factors have largely been neglected. Thus, this research highlights the importance of more factors in shaping and changing the language teaching cognition of EMP or ESP teachers in large, especially the teacher belief factor. In addition, results of independent samples *t*-tests indicated significant difference in EMP teacher learning in terms of gender, differences in EMP teacher attitude and teacher support in terms of EMP teaching experience. Suggestions for enhancing EMP TC are offered on the basis of the conclusions of this research.

## Introduction

Specificity has been described as the cornerstone of English for special purposes (ESP; Hyland, 2015). The teaching of English for occupational or professional purposes, such as English for medical purposes (EMP), has by nature been highly specific, since ESP teachers are expected to be specialists who “often need to be analysts first and foremost, then designers and implementers of specialized curricula” (Belcher, 2006, p. 136). It is a challenging role that involves multiple tasks, and teaching ESP is thus a demanding area in which to work (Basturkmen, 2012). ESP teachers often need to work independently on course design and materials development, which requires them to be familiar with the English language that used in specific fields and make up for gaps in their subject matter knowledge. This is particularly true of the ESP teachers in medical universities, or the so called EMP teachers. Though EMP teachers are not demanded to start their careers with a medical degree, general knowledge of the medical field is indispensable for developing teaching materials and classroom activities to meet learners’ needs, especially those who are eager to acquire and develop the literacy skills that will grant them access to the globalized medical community of practice (Agudo and Dios, 2014).

In recent years, the topic of ESP teachers and the teaching of ESP has begun to attract some interest in the literature (Ding and Bruce, 2017), unfortunately, few studies have addressed this issue in an empirical manner, and research on EMP teachers is even scarcer. Consequently, little is known, for example, about EMP teachers’ beliefs, how EGP teachers transition to EMP teaching, the challenges they confront in so doing, and the assistance they receive. The background of the current study is the large-scale reform of college English teaching in China, which centers on shifting from ELT to school-based, specific English teaching. Our interest in EMP teachers springs from three sources. First, English is the worldwide prime vehicle for the diffusion of medical knowledge, posing a major challenge for ESL/EFL learners, as they will need to acquire and/or develop key competences in English that are mainly connected with “not only the register of medical terminology, but also the preferred choices of lexis and syntax, typical discourse structures” (Maclean and Maher, 1994, p. 2431). Due to the wide use of EMP in medical and biological fields, EMP teaching has been vigorously advanced in more than 100 medical universities or faculties in China (Li, 2019), EGP-to-EMP transitions are thus consistently encouraged. Second, EMP teachers are involved in the design and delivery of both curriculum and instruction guided by ESP related theories, which deviates from the general patterns of EGP teaching, a domain that EMP teachers originally come from. Third, EMP teachers often find themselves in such a predicament that they too have to learn the language of medicine, to acquire and develop certain skills, knowledge, beliefs and attitudes before being able to design and teach EMP courses suited to learners’ specific needs (Tweedie and Johnson, 2019), leading to the constant rebuilding of EMP teachers’ knowledge repertoire, as well as their identities.

Thus, the aim of this study was to explore EMP teacher cognition (TC) concerning the qualities of an EMP teacher, efforts made in the EGP-to-ESP transition, and the development of new conceptions, whether about teaching as an activity or about the teachers themselves, or, in short, EMP TC of how to be a good English teacher at a medical institution.

## Literature review

### English for medical purposes teacher cognition constructs

Teacher cognition, “the unobservable cognitive dimensions of teaching – what teachers know, believe, and think” (Borg, 2003, p. 81), has attracted wide interest of research since the 1970s (Johnson, 2009). The literature on TC indicates that it shapes teachers’ framework for practice and affect their actual classroom activities (Borg, 2003, 2016), besides, TCs and practices are mutually informing with contextual factors playing an important role (Borg, 2003). TC currently has been so widely used as an umbrella term, and research on it covers the themes of teachers’ attitudes, identities, and emotions (Borg, 2009, 2012); beliefs (Burns, 1992; Richards, 1998); knowledge (Freeman, 2001); conceptions of teaching (Freeman, 1996); and conceptions of practice (Gitsaki and Alexiou, 2015) etc.

Studies examining what ESL/EFL teachers, at any stage of their careers, know, think, or believe with respect to various aspects of their work is a rather important part of TC studies (Kubanyiova and Feryok, 2015). Literature of research on ESL&EFL language TC is as extensive as mentioned above, covering the topics as diverse as teachers’ learning experiences (Farrell, 1999; Hayes, 2005); TC development (Tsui, 2003); roles in teaching practices (Borg, 1999; Basturkmen et al., 2004); teacher anxiety and supports from environments (Liu et al., 2022); cognitions develop by comparing inexperienced and experienced language teachers (Nunan, 1992; Richards et al., 1998); cognition in relation to language skills instruction (Zhao and Zhang, 2022, p. 48), etc.

Research on ESP TC is severely limited, as only a few studies have addressed ESP/EAP teaching methodology, EAP TC, and teacher education (Ding and Campion, 2016; Atai and Taherkhani, 2018). Such as how EAP teachers conceptualize and practice the four language skills, and the relationship between ESP TC and practice has also been tapped on (Baleghizadeh and Shakouri, 2017). In the research mentioned earlier, ESP TC was broadly defined as largely the same as EFL/ESL TC, i.e., an inclusive and multidimensional term that encapsulates the complexity of the mental lives of language teachers, including the beliefs, anxieties, attitudes, and identity of ESP teachers, as well as interaction with the environment and the teaching practices (Edwards et al., 2002).

The literature review highlights that TC is a category with multiple dimensions and that it is sometimes context-specific (Farrell and Ives, 2015). The cognition of Chinese EMP teachers, an important group of ESP practitioners in Chinese universities, may have characteristics similar to the TC of EFL/ESL/ESP teachers mentioned in the literature review. Therefore, the EMP TC constructs in this study initially include such variables as teacher attitude, teacher belief, teacher identity or teacher roles, teacher support, and teacher practice, with the purposes of exploring, validating, and constructing an EMP TC scale.

### Measuring English for medical purposes teacher cognition

The term ‘TC’ refers to the unobservable cognitive dimension of teaching: what teachers know, think, and believe. Education researchers have realized the importance of the mental lives of teachers as unique and active decision makers, leading to studies of the psychological dimensions of teaching (Borg, 2003). Since teachers’ mental constructs are unavailable for direct observation, various elicitation instruments such as standardized surveys containing categorical belief/knowledge statements or carefully developed interview guides and stimulated recall protocols are used for ‘mining’ teachers’ thoughts. Previous studies of TC of teaching EFL/ESL as well as ESP elicited explicit statements through interviews (Alexander, 2012; Kubanyiova and Feryok, 2015; Donald, 2016), surveys/questionnaires (Seferoǧlu, 2006; Maria, 2011; Nazari and Atai, 2022), or a combination of the two (Tsui, 1996; Richards et al., 1998; Mullock, 2006; Tatzl, 2013; Golombek, 2015). More recent studies have also used journal/diary studies (e.g., Kumazawa, 2013) and language portraits (e.g., Nishino, 2012) to elicit teacher’s beliefs.

From the literature review, it is apparent that in terms of methodology, most previous studies adopted qualitative case studies and/or interviews to investigate such factors of TC as teacher beliefs and attitudes. Several studies involved a questionnaire survey, including those conducted by Akbari and Tajik (2009) and Faez and Valeo (2012). Building on the insights provided by these previous studies, a quantitative study with a large sample size can contribute significantly to further exploring these issues and verifying the findings of previous research. Because EMP teaching is an integral component of ESP teaching—and more broadly—also a component of EFL/ESL teaching, the research methodologies adopted in the previous studies should be equally applicable to the exploration of EMP TC in this study. As stated in the opening paragraph of this section, EMP TC is defined and extensively recognized as a multidimensional construct comprising elements such as teacher attitude and teacher beliefs (Woods, 1996; Lee, 2008). Hence, a large-scale questionnaire survey would be a reasonable choice as a measurement tool. Therefore, in this study, the dimensions of EMP TC were measured by initially addressing the following variables: teacher attitude, teacher belief, teacher identity/roles, teacher support, and teacher practice.

### Teacher characteristics influencing English for medical purposes teacher cognition

Teacher cognition is a schema that encompasses multiple dimensions of the teachers and surrounding environments and is also subject to the differences in intrinsic factors between teachers, such as age, gender, work experience, and the institutions at which they work. Relevant research in the field of EFL/ESL TC is rather abundant, with the factors of teacher gender and teacher work experience attracting much greater attention. For example, Ross (1998) and Edwards et al. (2002) found that the construct of teacher efficacy interacts with teacher characteristics, such as gender and teaching experience. There are also studies covering the correlation between teacher gender and students’ in-class performance (Lau et al., 2005; Gentile et al., 2009; Adekola, 2010; Butucha, 2013; Cameron et al., 2013; Klassen and Tze, 2014; Li et al., 2015; Dror and Shoshana, 2022), levels of teacher self-efficacy between male and female gender groups (Tschannen and Woolfolk, 2001; Shi, 2009; Wright, 2010; Kang and Cheng, 2014; Shroyer et al., 2014; Dilekli, 2015; Zee and Koomen, 2016). The issue of gender difference in EFL/ESL teacher’s use of information technology (IT) is frequently noted in the literature too. Some findings indicate that male teachers, compared to female teachers, perceive themselves as more competent to analyze and reflect on data using IT and to produce digital content (Choi and Barbara, 2009; Xu et al., 2020). It is also of particular interest to identify potential gender differences in teachers’ beliefs, as they may determine teachers’ intention to use and the actual integration of new theories and technologies into classrooms (Ong and Lai, 2006; Tondeur et al., 2008; Teo, 2014). In addition, professional seniority or teaching experience was found to be a significant variable in teachers’ self-efficacy and belief for teaching (Instefjord and Munthe, 2016; Macia and García, 2016; Alarcon and de Vicente-Yagüe, 2020; Allen et al., 2020; Graham et al., 2020; Miguel et al., 2020). Teachers having low professional seniority are found to have higher self-efficacy than the teachers having high professional seniority (Yoon, 2002; Tschannen and Hoy, 2007; Shapiro, 2010; Roffey, 2011; Wang, 2015; Graham et al., 2020).

The literature review implies that TC indeed correlates with several factors of teacher characteristics, and should thus not be discussed in isolation. In considering the relationships between teacher belief, teacher attitude, teacher identity, organizational support, and teacher practice, we must also take into account the personal characteristics of the teachers, as previous studies indicate that EFL/ESL TC is sensitive to such individual and contextual factors (Bogler and Somech, 2004; Alexander, 2012; Aslrasoulia and Vahid, 2014; Liu et al., 2016).

Grounded in the theories and findings on TC, this study is designed to explore EMP TC at medical universities in China, centering on the interior and exterior factors of EMP TC. Furthermore, in this project, the potential links between several teacher characteristics and EMP TC, such as teacher age, gender, teaching experience, and titles, are also assessed. In doing so, this study addresses the following research questions:

(1) What is the structure of EMP TC?
(2) What are the levels of EMP TC?
(3) Are there any significant differences in EMP teachers’ cognition in terms of teacher characteristics such as gender or years of teaching?

## Materials and methods

### Participants

The participants were all EMP teachers from medical universities or polytechnic or comprehensive universities that award medical degrees in China. Of the 254 participants, 63 (24.8%) were male and 191 (75.2%) were female (see Table 1). Regarding working experience, 235 (92.5%) had served as a college English teacher for 5 years or more, with 191 (75.2%) for 10 years or more, indicating a group of practitioners with rich EFL teaching experience. When it comes to EMP teaching experience, 98 (38.58%) had been engaged in EMP teaching for less than 5 years, and 156 (61.42%) had been working in this field for 5 years or more.

**TABLE 1:** Exploratory factor analysis (EFA) results of the 31-item scale (pattern matrix).

Items	Factor 1	Factor 2	Factor 3	Factor 4	Factor 5	Factor 6	Commonalities
		
	Teacher	Teacher	Teacher	Teacher	Role	Teacher	
	attitude	belief	learning	support	identification	practice	
Q05 MET benefits students’ future career.	0.886						0.751
Q06 MET boosts the quality of talent cultivation.	0.825						0.721
Q07 MET meets the social demand.	0.820						0.657
Q04 MET enhances students’ skills.	0.815						0.728
Q03 MET is a part of English teaching reform.	0.669						0.553
Q13 MET strengthens my sense of professional belonging.	0.589						0.590
Q01 I am interested in MET.	0.547						0.447
Q02 I am a competent medical English teacher.	0.505						0.422
Q16 EMP teachers should focus on the market demand.		0.875					0.820
Q15 EMP teachers should be clear about the medical culture and values.		0.848					0.782
Q17 EMP teachers should know about the medical working environment.		0.754					0.772
Q14 EMP teachers should be familiar with the ESP theory.		0.682					0.534
Q18 EMP teachers should be clear about features of medical language.		0.634					0.666
Q19 EMP teachers ought to communicate with students about progresses in the medical industry.		0.553					0.591
Q48 I read books or journals on ESP research.			0.886				0.784
Q47 I browse the official account of ESP teaching and research.			0.878				0.762
Q49 I joined the virtual community EMP teacher development.			0.729				0.657
Q34 I exchange views with people in medical field over talent cultivation.			0.615				0.480
Q52 Online resources for medical English teaching are scarce.				0.948			0.897
Q51 Professional in-class teaching materials of medical English are hard to get.				0.714			0.532
Q53 Communication platforms for EMP teachers are rare.				0.633			0.443
Q21 EMP teachers should prepare lessons with medical professionals.					0.798		0.648
Q22 EMP teachers should conduct team teaching with medical professionals.					0.776		0.646
Q20 EMP teachers should have a wealth of medical knowledge.					0.492		0.486
Q24 EMP teachers should guide students in setting up learning goals.					0.435		0.510
Q37 I guide students to pay attention to cultural differences.						–0.739	0.626
Q38 I choose to have both formative and summative evaluation.						–0.665	0.596
Q36 I use self-compiled materials in class.						–0.615	0.501
Q44 I review and reflect on my teaching practices after class.						–0.585	0.517
Q33 I share with students the online learning resources.						–0.553	0.536
Q39 I know how and where to get answers when I am asked professional medical questions.						–0.511	0.520
Cumulative%	33.830	43.236	53.002	55.401	59.786	62.838	——
Reliability	0.901	0.836	0.875	0.893	0.905	0.878	——

MET, medical English teaching.

### Instrument

An item pool questionnaire was firstly built by referring to previous studies on ESL/EFL/ESP TC in both the Chinese and other contexts (Bojovic, 2006; Derya et al., 2016). Through the blending of information derived from this process, a draft of a 60-item questionnaire was completed. Three ESP professors with a teaching and research experience of 10 years or more jointly proofread and edited the questionnaire, and seven items were removed due to repetition of information or poor relevance.

The final “EMP TC” questionnaire consisted of five parts. The first part is about participants’ demographic information. The second part comprised statements to unveil EMP TC in terms of the participants’ attitude toward EMP teaching, cognition of an EMP teacher’s role, choices in EMP teaching and the efforts made beforehand, as well as the participants’ views on expected support in the transition to becoming a competent EMP teacher.

All the responses to statements in this questionnaire were based on a 5-point Likert scale, with 1–5 respectively representing “Strongly Disagree,” “Disagree,” “Neither Agree Nor Disagree,” “Agree,” and “Strongly Agree.”

### Data collection and analysis

The online questionnaire was administered in the second half of the 2021 academic year, from mid-September to the end of October, which was right at the beginning of a new semester for Chinese universities, lasting for around 6 weeks. A total of 254 questionnaires were identified as valid with complete information and valid responses. In analyzing the data, first, a dichotomous variable was introduced for teachers’ gender (0/male, 1/female), continuous variables were used to indicate teachers’ age (30–60), teachers’ total number of teaching year(s), and EMP teaching year(s). Second, the questionnaire survey results for the 31 extracted items were recorded (with each survey item having a rating between 1 and 5), calculated and merged using SPSS 26.0 software. This was then followed by descriptive statistics for the factors and levels of EMP TC (see Table 2). The results were listed by presenting the minimal value, maximum value, mean value, levels of standard deviation, and sequenced by the level of mean value. Thirdly, in order to explore the possible influence of teacher characteristics such as teacher gender, teaching experience on EMP TC, independent samples *T*-Tests were carried out using SPSS 26.0 software. The results were listed by presenting the mean value, *t* value, and *p* < 0.05 was set as statistically significant.

**TABLE 2:** Descriptive statistics of the levels of EMP teacher cognition.

	*Min*	*Max*	*M*	*SD*
Teacher belief	2.17	5.00	4.44	0.56
Teacher attitude	2.00	5.00	4.33	0.59
Role identification	2.25	5.00	4.22	0.62
Teacher practice	1.00	5.00	4.17	0.61
Teacher learning	1.00	5.00	3.59	0.92
Teacher support	1.00	5.00	3.48	0.94

*N* = 254.

An initial review of the questionnaire findings revealed that the participants had a clear understanding about the need for subject knowledge in EMP teaching. They invariably viewed this kind of knowledge as essential. However, challenges kept on spring up in the period of transition, which led to the dynamic changes and re-building of their beliefs. The detailed analysis and results are presented in the “Results and Discussion” section.

## Results and discussion

### Structure of the English for medical purposes teacher cognition

In order to address the first RQ, the exploratory factor analysis (EFA) was conducted to explore the dimensionality of EMP TC. The extraction method of principal axis analysis and the rotation method of direct oblimin were then adopted. The threshold of factor loading was set at 0.40. Following the ideas of Ho (2006), Hair et al. (2010), and Gao and Liu (2022), items with a factor loading lower than 0.40, items with cross-loadings and those were theoretically or logically inconsistent with other items under the same dimensions were deleted.

The Kaiser–Meyer–Olkin (KMO) (*KMO* = 0.901 > 0.70) and Bartlett’s test (*p* < 0.001) results were desirable (χ*^2^* = 5286.492, *df* = 465). In the analysis that followed, 22 items (items 8–12, 23, 25–32, 35, 40–43, 45, 46, 50) were discarded, which resulted in a 31-item scheme (see Table 1). Six factors were identified, accounting for 62.838% of the variance. The commonality of each item was greater than 0.42. The internal consistency of the global scale and the six factors involved were assessed, and the Cronbach’s alpha for the whole scale and six factors were, respectively, 0.895, 0.901, 0.836, 0.875, 0.893, 0.905, and 0.878, suggesting high reliability (Creswell, 2014).

Factor 1 contains a total of eight items concerning medical English teachers’ views of the significance of teaching medical English (items 4–7), the prospect of ESP-oriented English teaching reform (item 3), and whether they can be competent medical English teachers (items 1, 2, 13). This factor is labeled “teacher attitude” because, as Baker (1992, p. 261) stated, “language attitudes, an umbrella term for attitudes toward language and language learning and teaching are complex constructs composed of cognitive, affective and behavioral elements.” In this questionnaire, the eight items above successfully elicited the participants’ cognition of the value of teaching medical English and the teachers’ self-judgment. Such a teacher attitude evaluation is particularly necessary due to the declaration in the instructions of the *Guide of College English Teaching* (2020 Edition) in China that “courses of English for specific purposes, together with courses of English for general purposes and cross-cultural communications, are the major content of English teaching in college.” It suggested that ESP should be more widely advocated, especially when it corresponds with the major disciplines of the institutions. In medical universities, such a transformation thereby means that former English teachers of EGP should shift to the field of ESP, or EMP in particular (Liu and Xu, 2009; Choi, 2021). In psychological literature, beliefs are considered to influence attitudes, and while attitudes reflect evaluative dispositions, beliefs are assumptions about what is true or not (Eagly and Chaiken, 1993). To most teachers involved, the above transformation is full of uncertainties and challenges, but it also stimulates their desire to learn and explore. It is their attitude toward such a shift that shapes their choices and leads to subsequent actions.

Factor 2 involves teachers’ understanding of ESP theories and the related demand for teachers to have knowledge of medicine, including medical English application scenarios, language standards, cultural value differences (items 15–17, 19) that medical English teachers should pay attention to, as well as teachers’ opinions on medical English (Item 14, 18). The differences between ESP and EGP are mainly reflected in the aspects of genre, form, language function, etc. Medical English has distinct characteristics in terms of lexical composition, genre, and style that limit the scenarios of application (Elsted, 2012; Hyland and Jiang, 2021). Therefore, the planning and execution of learning activities and the evaluation of EMP courses are largely preconditioned by medical English teachers’ familiarity with ESP theories and their understanding of the medical field. Obviously, factor 2 covers both EMP teachers’ understanding and potential teaching move. According to Borg (2003, 2017), teachers’ beliefs are a key focus of the language TC research, in which beliefs are typically discussed in relation to teachers’ practices. Other scholars generally agree that there is a reciprocal but complex relationship (e.g., Mansour, 2009; Basturkmen, 2012) and beliefs and practices may influence one another depending on the context (e.g., Richardson, 1996; Liu et al., 2021). Thus, factor 2 is labeled “teacher belief.”

Factor 3 concerns teachers’ learning and professional development during and after the EGP-to-EMP transition. It involves both the explicit efforts individual medical English teachers make to learn about and research EMP teaching (items 47, 48) and the implicit efforts they make to engage with the medical environment and medical professionals or communities of ESP/EMP practitioners (items 34, 49). It should be noted that English language educators are commonly equipped with teaching pedagogies and knowledge of the English language to teach in school contexts (Zeichner, 2005; Kabilan and Izzaham, 2008). They are generally taught about the principles, theories, and practices that prepare them to teach EGP (Ong et al., 2004; Evans and Esch, 2013). Dudley-Evans (1997) argued that learners’ “specialist knowledge” is a major factor that distinguishes ESP teaching from ESL/EFL teaching, as medical English teachers are “not in the position of being the ‘primary knower’ of the carrier content of the material.” However, Ferguson (2007) deliberately chose the term “specialized knowledge” to contrast with “specialist knowledge,” which is usually understood to refer to knowledge of the content of the student’s discipline or subject. To him, specialized knowledge involves three related elements: (a) a sociological knowledge of disciplinary cultures and values; (b) a philosophical knowledge of the epistemological bases of different disciplines; and (c) a linguistic knowledge of genre and discourse. As social, ecological, and socio-cognitive factors influence what teachers learn (Molle, 2021), no matter how professional the medical knowledge might be, for most English teachers whose expertise lies elsewhere, there would be an arduous journey of learning demanding tremendous courage and lasting effort. Therefore, it is appropriate to label factor 3 “teacher learning.”

Factor 4 refers to the material and non-material assistance that medical English teachers hope to get from the school environment, including assistance in obtaining accessible and authoritative materials for medical English teaching or research as well as online resources (items 61, 62) and platforms for communicating and sharing both intellectually and emotionally (item 63). According to McDonald (2012), educational reform requires teachers to adapt or change their belief systems and educational practices during the implementation process. Oplatka (2018) argued that educational reform is complex and the implementation of educational reforms results in changing the *status quo*, propelling teachers’ enthusiasm to change. Despite the possible disadvantages of borrowed reforms, teachers are asked to adapt or change their belief systems and educational practices to implement the mandated change (Romanowski and Du, 2020), which is exactly the case of the college English reform taking place in Chinese universities currently. To ESP/EMP teachers in universities, support for teaching and research generally comes from the school environment, and in a narrow sense, social support is regarded as resources provided by others, such as assistance in coping with new conditions or an exchange of resources (Pourfeiz, 2013). Correspondence of the loadings of factor 4 are apparent in Weiss’s (1974) theoretical model of social support—namely, guidance (advice or information), reassurance of worth (recognition of one’s competence), and social integration (a sense of belonging to a group of friends). On the basis of the above, factor 4 is labeled “teacher support.”

Factor 5 involves medical English TC of EMP teaching as a whole, with items 21 and 22 relating to the teachers’ understanding of the roles in team teaching and item 24 to their understanding of the role in instructing students, which are in line with the five defined roles of an ESP teacher as teacher, collaborator, course designer and materials provider, researcher, and evaluator (Dudley-Evans and John, 1998). Item 20 relates to medical English TC of the importance of professional knowledge of medical English, which is important for the teachers’ role shift, since English language educators need to move from teaching general English to teaching English in a specific context in teaching ESP (Basturkmen, 2012; Liu and Chu, 2022). It also means that English teachers are expected to have medicine-specific knowledge and skills before they engage in teaching EMP. Through collaboration and cooperation with medical professionals, EMP teachers can exchange ideas and experiences with them to deal with medicine-related texts and tasks, and thus equip themselves with the necessary knowledge and tools. Some scholars have even pointed out that team teaching or cooperation with content teachers is distinctive to ESP (Richards and Farrell, 2005), though ESP teachers must reconsider their roles or even reconstruct their professional identities. Thus, it is reasonable to label factor 5 “role identification.”

Factor 6 relates to the actions medical English teachers take at three stages: (a) preparation before class, (b) organizing and teaching in class, and (c) evaluation after class. Item 39 relates to the stage of course preparation, where medical English teachers have to prepare for potential challenges due to a lack of medical expertise. Items 33, 36, and 37 relate to the different aspects on which medical English teachers focus while teaching, such as guiding students about the cultural differences they might come across in future work (item 37), since one of the objectives of ESP teaching is “getting students to prepare for the cultural variations” (Yu et al., 2017). As a facilitator and resource provider (Dudley-Evans, 1997), an important job of the EMP teacher is to guide students in developing competence in searching independently for appropriate learning resources and prepare appropriate course materials for classroom activities (items 33, 36). It is equally important to cultivate students’ sensitivity to the styles of academic publications (Li and Flowerdew, 2020), enhancing their competence in genre analysis (Huttner et al., 2009). Item 38 relates to the evaluation of student learning. Unlike in traditional EFL/ESL teaching, in EMP teaching, students’ learning needs are more immediate and career-oriented (Basturkmen, 2008; Jiang et al., 2016; Choi, 2021), which leads to a significant change in the way of evaluation, which focuses heavily on students’ learning process (Fox, 2009). Item 44 relates to teachers’ reflection after class, since teacher reflection is not only a necessary skill for EMP practitioners, but it also contributes significantly to the students’ professional development (Basturkmen, 2012; Lazarević, 2022). Thus, factor 6 is labeled “teacher support.”

### Levels of English for medical purposes teacher cognition

As is shown in Table 2, of the six factors of EMP teacher cognition, teacher belief achieved highest mean score (*M* = 4.44; *SD* = 0.56), followed by teacher attitude (*M* = 4.33; *SD* = 0.59), role identification (*M* = 4.22; *SD* = 0.62), teacher practice (*M* = 4.17; *SD* = 0.61), teacher learning (*M* = 3.59; *SD* = 0.92), and teacher support (*M* = 3.48; *SD* = 0.94).

The mean value of the first four factors of EMP teacher cognition listed in Table 2 (teacher belief, teacher attitude, role identification, and teacher practice) all have scores of above 4, which is much higher than the scores for the last two factors (teacher learning and teacher support). This difference might be ascribed to the fact that the first four factors are related to the EMP teachers themselves, or that they are more subject-specific. It is therefore clear that ESP (EMP here) teachers’ cognition of language teaching is significantly influenced by intrinsic factors (Johnson, 2009). Against the backdrop of the grand reform of foreign language teaching in Chinese universities, most English teachers clearly recognize the limitations of traditional EGP-centered teaching (Cai, 2015), while the necessity and urgency of the EGP-to-ESP transition has been emphasized by and again at EFL teaching reform meetings at all levels. In such an ESP-friendly context, English teachers at medical universities and institutions are fully prepared to accept and reinforce their identities as EMP teachers. They have a highly positive understanding—or otherwise—of what a transition to ESP teaching entails (Ferguson, 2007), and still have good reason to remain positive about the prospects of EMP teaching as well.

The last two factors are closely linked to the environment or are highly context dependent. On the one hand, ESP teaching is gaining significant traction at many institutions in China. On the other hand, however, due to the inflexible patterns of the pedagogical system, fixed standards of teacher evaluation that cater exclusively to the conventional teaching of EGP, and limited ESP teaching resources, EFL teachers have doubts, feel uncertain, or even antagonism toward their prospects of successfully transitioning into an ESP teacher. However, ESP teachers must deal with significant challenges (Tao and Gao, 2018), such as working with subject teachers who might have opposed pedagogical beliefs. The transition from general English to ESP/EMP is also a journey on which “teachers’ learning never ends” (Campion, 2016), as ESP/EMP teachers must surmount subject matter knowledge gaps (Wu and Badger, 2009; Atai and Nejadghanbar, 2017). Therefore, EMP teachers need to be assured of firm and sustainable support from the institutions. However, the responses from participants in this study indicate that EMP teachers’ vast investment in knowledge restructuring, new curriculum designing and execution, or disconnection from previous teaching and researching norms receive insufficient attention, or are even ignored. Such facts may very well explain the relatively few initiatives for EMP teacher learning at some institutions and the low level of recognition or support they receive from schools.

### Gender differences and English for medical purposes teacher cognition

A t-test was run to investigate any specific relations between teacher gender and EMP teacher cognition. The results indicate a significant difference in teacher learning in terms of EMP teachers’ gender (*t* = 1.654, *df* = 252, *p* < 0.05).

Compared with female EMP teachers, the male participants hold a more positive attitude toward teacher learning. Gender might have been less of a focal point in previous studies on teacher learning, but the variables of teacher cognition and beliefs, teacher emotions, teacher motivation, and context account for a lot in teacher learning (Pinar et al., 2021). EGP teachers are likely to confront various difficulties when they switch to ESP/EMP teaching, and their biggest challenge is a lack of subject knowledge. Campion (2016) found that ESP teachers at the initial stage were low in confidence, and the entire group was often overwhelmed by this challenge, making them resistant to subject knowledge. Because the EMP teachers in this study were predominantly transferred EGP teachers, they also had to address the challenge of medical knowledge deficiency. In fact, EMP teachers are not only pressured by the subject knowledge issue but also have to deal with potential distrust from students and subject teachers. In this study, EMP teacher learning included reading ESP books or journals, following and browsing official accounts of academic organizations, joining online ESP or subject knowledge learning communities, and engaging with medical professionals.

It must be emphasized that such learning activities involve both technical and interpersonal elements, in addition to literacy activities. Keskin and Yazar (2015) reported that male teachers had a higher ability to use computers and acquire digital media information faster than female teachers. Yoon (2022) also demonstrated that male and female teachers differ significantly in several aspects of digital competence. Male teachers are more willing than female teachers to adopt new technology for work. In addition, male teachers tend to be more active in environments involving interpersonal communication and cooperation (Aslrasoulia and Vahid, 2014). ESP/EMP teaching is neither a simple language course nor a simple subject course but is a highly integrated course with equal emphasis placed on language use and subject knowledge (Murray, 2019). In the initial stages of EMP teaching, teachers are encouraged to develop the habit of reflecting on whatever happens in class, especially on accidental events associated with subject knowledge. They are also advised to carefully study responses from students to identify the knowledge gap between the needs of their students and what they can deliver in class, which often requires both subject knowledge and ESP skills. When the teaching materials involve specific medical information, novice EMP teachers are typically puzzled by questions such as: “What are the major theories in this field?”, “What are the basic medical principles involved?”, and “How far should I go with the subject knowledge in class?”. Such questions are by no means easy for language teachers who have not received systematic medical education. A major approach via which EMP teachers can make up for such a knowledge gap would be to consult medical professionals. In such a case, EMP teachers can seek assistance from medical professionals, summarize the main medical points involved with the professional help, or even visit medical departments to get a close-up look at the operational procedures themselves. When such a need for communication arises, female EMP teachers are more likely to be resistant to going so far as having direct contact with medical workers or exposure to medical scenarios.

### Differences in years of teaching on English for medical purposes teacher cognition

Another t-test was run to investigate potential relations between fears of EMP teaching and EMP teacher cognition. The results show a significant difference in the impact of EMP teaching experience on teacher attitude (*t* = −3.807, *df* = 248, *p* < 0.05) and teacher support (*t* = 0.447, *df* = 248, *p* < 0.05).

The participants in this study were all from medical universities or polytechnic or comprehensive universities that offer medical degrees, which is a distinct fact as “EFL teachers from medical, technological universities and alike are more advantaged and better equipped groups in carrying out ESP teaching than counterparts from institutions of other types” (Cai, 2015, p. 96). Five years of EMP teaching was used as a threshold level in this study, since it has been suggested that teachers experience significant mastery progression especially in the early stage of their career (first 5–10 years of teaching experience) (Rice, 2013). The results indicate that teachers with EMP teaching experience of 5 years or more, who accounted for 61.42% of the total sample, demonstrated a more positive attitude (*p* < 0.05). Teacher expertise and teaching experience are often used in parallel despite their differences, and the common assumption is that the length of teaching experience promotes teachers’ effectiveness, knowledge, and skills (Rice, 2013; Muhonen et al., 2021). As they gain teaching experience, EMP teachers become more familiar with the theories in this field, gain a clearer understanding of students’ EMP learning, and receive warmer responses from students, which will, in turn, promote their teaching confidence and strengthen their positive attitude.

Regarding teacher support, the results suggest that EMP teachers with longer teaching experience pay significantly more attention to teacher support than teachers with less than 5 years of EMP teaching experience (*p* < 0.05). Some of the results from this study also suggest that teacher support should initially come in the form of explicit assistance from the organization. For example, authoritative EMP publications currently serve as a valuable resource pool for EMP teachers. With such materials, language teachers are guided through the focal points concerning subject knowledge. Furthermore, these materials are generally context-based, with good examples illustrating the language characteristics and impelling practice of the communicative skills in English used in medical environments. Medical journals, which update medical trends and research fronts, help enhance the medical literacy of EMP teachers and provide a wealth of real corpora for medical English teaching. In addition to explicit support for teaching facilities, teacher support also includes implicit support (e.g., teaching teams and communication platforms). “Compared with EGP teaching, although EMP and ESP teaching resources are still relatively scarce, the existing teaching materials, such as textbooks and teaching software, can basically meet the requirements of EMP teaching” (quote from Mr. Yin, associate professor at a medical university who participated in this study). Seniority is a conspicuous contributing factor to teachers’ need for support with professional development. School-based environmental resources, such as supportive supervisors and colleagues (Kokkinos, 2007; Droogenbroeck et al., 2014), play a central role in improving teaching performance and serve as effective initiators of employee work engagement (Xanthopoulou et al., 2009). Kirkgöz and Dikilitaş (2018) suggests that ESP training programs are another source of immediate assistance; in most cases, at present, professional ESP practitioners train themselves, learning as they progress. Currently, the selection of training programs designed for ESP teachers in China continues to grow (Lin, 2018). These programs generally comprise ESP methodology teaching and workshops practicing ESP teaching in specific fields of expertise, such as medicine, law, or engineering. In such a disciplinary culture, trainees benefit from communication and learn through decision-making practices, which contribute to their conceptualization and practice of appropriate notions for ESP lessons.

## Conclusion

Through a large-scale questionnaire survey and EFA, this study explored and built up a 6-factor structure of EMP teacher cognition, including teacher attitude, teacher belief, teacher learning, teacher support, role orientation, and teacher practice. This study also confirmed the influence of gender as a factor influencing EMP teacher learning, and the influence of teaching experience on EMP teacher learning and support. The results of this study may shed light on ESP teacher research in the following respects.

The scale of EMP teacher cognition has been theoretically confirmed and has extended the findings of prior studies on both EFL/ESL and ESP teacher cognition. Factors such as teacher belief, teacher attitude, teacher identity (labeled role identification in this study), and teacher practice have been confirmed to be the most significant elements of EMP teacher cognition. However, in this study, EMP teacher learning and teacher support also emerged as crucial factors, which is novel to the umbrella of teacher cognition. Such new findings validate the assumption that teacher cognition is a multidimensional, complex concept that is context-dependent in this study. On the other hand, the research findings demonstrate the specificity of EMP teachers, who are a group of practitioners serving as teachers, collaborators, course designers, materials providers, researchers, evaluators (Dudley-Evans and John, 1998)—and most importantly—active contributors to EFL teaching reform in this research. This finding inspires us with reassurance that future studies can further validate the current structure of EMP teacher cognition and explore in depth the characteristics and complexity of ESP teacher cognition in large, expanded samples and a broader variety of participants.

Previous studies on language teacher cognition have already established the significance of factors such as teacher belief, attitude, identity (roles), and the reciprocal effect of teacher practice. This study contributes new findings concerning the significant effect of teacher learning and teacher support on EMP teacher cognition, which demonstrates not only the multidimensional nature of EMP teacher cognition, but also its complexity. Furthermore, this finding implies that medical universities and institutions, as well as policy makers involved in EMP teaching, should take concrete steps to facilitate EMP teacher learning and establish a more favorable environment for EMP teacher development. The steps to be taken include a consistent supply of authoritative EMP publications to serve as a resource pool, offering ESP-specific or school-based in-service training (SIT), and building up a comprehensive and flexible ecosystem, as proposed by Cai (2015). ESP teachers in China often experience a loss of identity as they work hard to meet the standards and become competent ESP practitioners. The teacher evaluation system is by no means ESP-friendly, as it has remained rigid and largely unchanged. Because teacher cognition and teacher practice are reciprocal to each other, a more favorable EMP teaching effect can be expected when EMP teacher development is realized with adequate external support.

## Data availability statement

The original contributions presented in this study are included in the article/supplementary material, further inquiries can be directed to the corresponding authors.

## Ethics statement

The studies involving human participants were reviewed and approved by the School of Foreign Languages, Hubei University of Chinese Medicine. The participants provided their written informed consent to participate in this study. Written informed consent was obtained from the individuals for the publication of any potentially identifiable data included in this article.

## Author contributions

ZKC designed the study, carried out data collection and analysis, and wrote the first draft of the manuscript. ZHZ and YL revised the manuscript. LPP contributed a lot in streamlining the interview data analysis, and she also brought in a new perspective to the revision of the discussion and suggestion sections. All authors agreed to the final version.
